# Detection of the carbapenemase gene *bla*_VIM-5_ in members of the *Pseudomonas putida* group isolated from polluted Nigerian wetlands

**DOI:** 10.1038/s41598-018-33535-3

**Published:** 2018-10-11

**Authors:** Olawale O. Adelowo, John Vollmers, Ines Mäusezahl, Anne-Kristin Kaster, Jochen A. Müller

**Affiliations:** 1 Department of Environmental Biotechnology, Helmholtz Centre for Environmental Research - UFZ, Leipzig, Germany; 2 Environmental Microbiology and Biotechnology Laboratory, Department of Microbiology, University of Ibadan, Ibadan, Nigeria; 3 Institute for Biological Interfaces (IBG5), Karlsruhe Institute of Technology, Eggenstein-Leopoldshafen, Germany

## Abstract

There are increasing concerns about possible dissemination of clinically relevant antibiotic resistance genes, including genes encoding for carbapenemases in the environment. However, little is known about environmental distribution of antibiotic resistance in Africa. In this study, four polluted urban wetlands in Nigeria were investigated as potential reservoirs of carbapenem-resistant bacteria (CRB). CRB were isolated from the wetlands, characterized by Blue-Carba test, MIC determinations and whole genome sequencing (WGS). Nine of 65 bacterial isolates identified as members of the *Pseudomonas putida* group (*P*. *plecoglossicida* and *P*. *guariconensis*, respectively) harboured the metallo-beta-lactamase gene *bla*_VIM-5_. WGS revealed the *bla*_VIM-5_ in three novel Tn402-like class 1 integron structures containing the cassette arrays *aadB*|*bla*_VIM-5_|*bla*_PSE-1_, *aadB*|*bla*_VIM-5_|*aadB*|*bla*_PSE-1_, and *bla*_VIM-5_|*aadB*|*tnpA*|*bla*_PSE-1_|*smr2*|*tnpA*, respectively. Strains carrying the *aadB*|*bla*_VIM-5_|*bla*_PSE-1_ cassette also carried an identical integron without *bla*_VIM-5_. In addition_,_ the strains harboured another Tn402-like class 1 integron carrying *bcr*2, several multidrug resistance efflux pumps, and at least one of *ampC*, *aph*(3”)-lb, *aph*(6)-ld, *tetB*, *tetC*, *tetG*, *floR*, and *macAB*. This is the first report of a carbapenemase gene in bacteria from environmental sources in Nigeria and the first report of *bla*_VIM-5_ in environmental bacteria isolates. This result underscores the role of the Nigerian environment as reservoir of bacteria carrying clinically relevant antibiotic resistance genes.

## Introduction

Antibiotic resistance is an increasing global problem with significant impact on human health^1^. This problem is aggravated by the emergence of resistance in the natural environment^2–5^. The increasing global detection of carbapenemase genes in bacteria from non-clinical sources^6–14^ is a disturbing observation that coincides with the recognition that natural ecosystems affected by anthropogenic pollution might play a role in the evolution and dissemination of clinically important antibiotic resistance^2–5^. This scenario point to the need for extended surveillance of non-clinical sources as reservoirs of bacteria harbouring genes encoding resistance to carbapenems for clinical and public health safety^14–16^.

Aquatic ecosystems are particularly important as conduits for the dissemination of antibiotic resistant bacteria and antibiotic resistance genes into other environmental compartments and eventually the human population^17^. Thus far, very few studies have investigated African aquatic ecosystems as reservoir of bacteria carrying antibiotic resistance genes of public health interest^18–20^ and none that we know of has investigated the presence of carbapenemase genes in environmental bacteria isolates in Nigeria, the most populous nation in Africa. Reports of carbapenemase genes in bacteria from Nigeria have been limited to clinical bacteria isolates^21–25^. The aforementioned studies reported detection of the carbapenemase genes *bla*_OXA-23_, *bla*_OXA-48_, *bla*_OXA-181_, *bla*_NDM-1_, *bla*_GES_, *bla*_VIM-1,_ *bla*_VIM-2_ and *bla*_KPC_ in clinical bacterial isolates. In addition, Walkty *et al*.^26^ reported the isolation of a *Pseudomonas aeruginosa*, *Klebsiella pneumoniae* and *Escherichia coli* carrying *bla*_VIM-2_, *bla*_NDM-1_ and *bla*_OXA-181_ respectively in a patient admitted to a Canadian hospital after prolonged hospitalisation in Nigeria. Only recently, Kazmierczak *et al*.^27^ reported the detection of the carbapenemase gene *bla*_VIM-5_ in clinical isolates of *Proteus mirabilis and Pseudomonas aeruginosa* from Nigeria, however, no information was provided about the genetic context of the genes. More importantly, very little is known about the genetic platforms linked to detected carbapenemase genes in Africa^28,29^. These are important omissions considering the widespread and uncontrolled use of beta-lactam antibiotics in human clinical therapy and food animal production in Nigeria^30^, and the ubiquitous release of untreated wastewater from several point and non-point sources into the aquatic ecosystem as a result of poor sanitation^31^. These predisposing factors for the proliferation of antibiotic resistance in environmental reservoirs make the investigation of Nigerian aquatic ecosystem as reservoir of carbapenemase-producing bacteria an urgent task.

The objective of this study was to investigate four polluted urban wetlands in Nigeria as potential reservoirs of bacteria harbouring genes encoding resistance to carbapenems. Isolated carbapenemase-producing bacteria species were subjected to WGS to understand the genetic platforms linked to detected carbapenemase genes. The results provide further insight into the global epidemiology of this important group of antibiotic resistance genes.

## Results

### CRB were recovered from urban Nigerian wetlands

**Figure 1:**
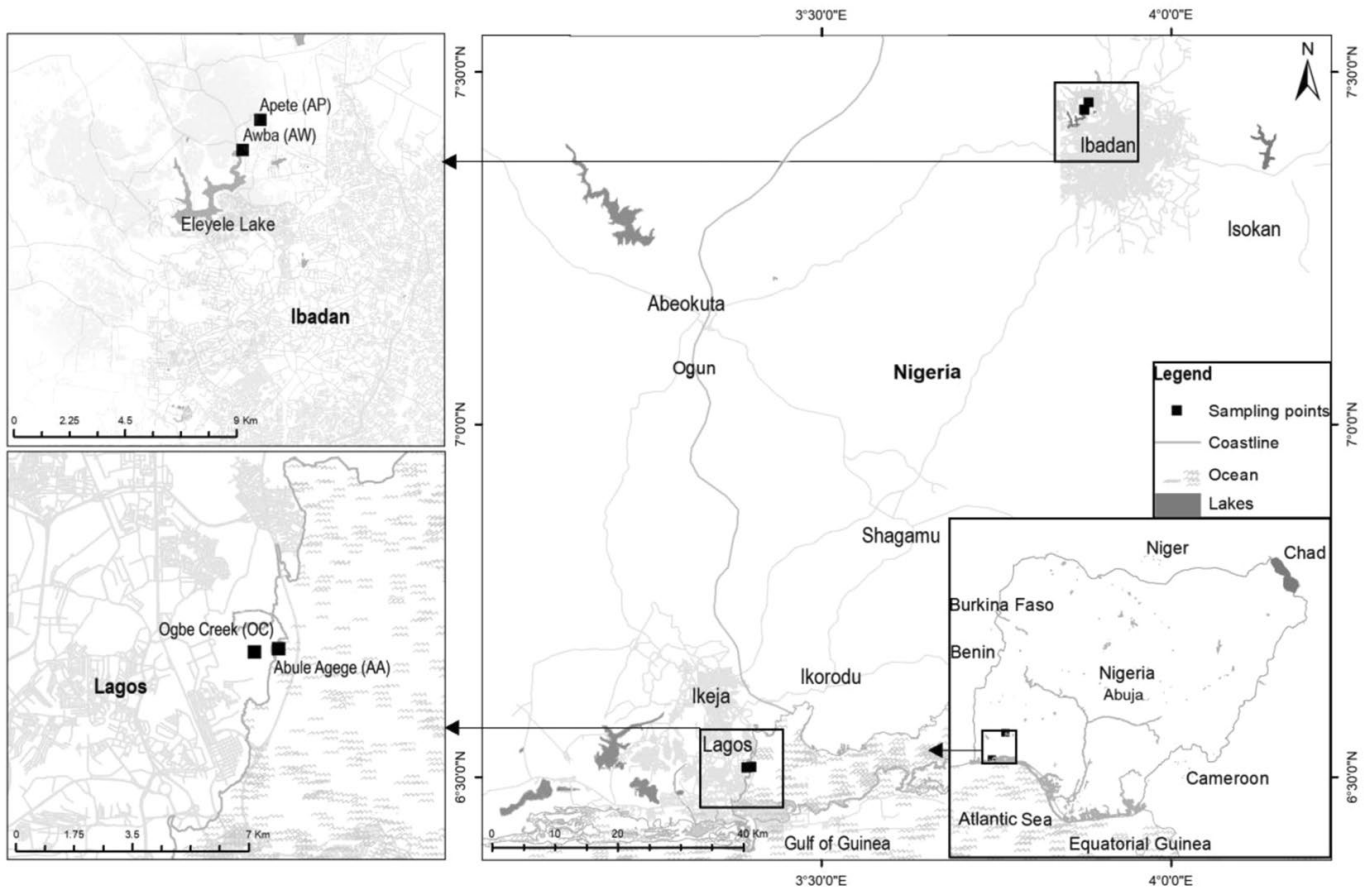
Location of sampling sites, indicated by filled squares (The map was created by Muhammad Arslan).

Various colonies of bacteria showing resistance to meropenem were isolated on all agar types: Muller Hinton (MH), Eosine Methylene Blue (EMB), and Pseudomonas isolation (PI) agar used for bacteria isolation from all sediment samples analysed. A total of 65 isolates showing different colony morphologies were isolated from Awba (AW = 14) and Apete (AP = 22) wetlands in Ibadan, and from Abule Agege (AA = 14) and Ogbe Creek (OC = 15) in Lagos (Fig. 1). According to their partial 16S rRNA gene sequences (about 1,300 bp) the strains belonged to six different genera (relative frequencies in parenthesis), namely *Pseudomonas* (52.3%), *Stenotrophomonas* (15.3%), *Cupriavidus* (12.3%), *Burkholderia* (9.2%), *Pandoraea* (6.2%) and *Ralstonia* (4.6%). Carbapenemase activity was detected by the Blue-Carba test in 61 isolates (93.8%) with at least one representative of each genus among the carbapenemase-producing strains. The isolates negative in the Blue-Carba test belonged to the genera *Cupriavidus* (3 isolates from AP in Ibadan) and *Ralstonia* (1 isolate from OC). Members of the *Enterobacteriaceae* were not present among the cultured CRB as deduced by absence of lactose-fermenting colonies on EMB agar plates. However, other *Enterobacteriaceae* (*E*. *coli*, *Enterobacter* spp., *Citrobacter* spp.) with resistance to third generation cephalosporins, fluoroquinolones and sulphonamides were isolated from the same samples in an accompanying study (Adelowo *et al*., unpublished), which suggests that there were indeed only few, if any, cultivable carbapenem-resistant *Enterobacteriaceae* at the sites.

### *bla*_VIM-5_ was detected in isolates belonging to the *Pseudomonas putida* group

The metallo-beta-lactamase (MBL) gene *bla*_VIM-5_ was the only carbapenemase gene detected of all carbapenemase genes (*bla*_VIM_, *bla*_KPC/BIC_, *bla*_NDM_, *bla*_IMP_, *bla*_SPM_, *bla*_AIM_, *bla*_DIM_) screened for by PCR among the 61 carbapenemase-positive isolates. This gene was found in nine isolates (prevalence rate of 15%) from AW and AP (2 samples each). The gene shared 99% nucleotide sequence identity with *bla*_VIM-5_ on the class 1 Integron of *Enterobacter cloacae* SMART 28 (accession number LC169578). The search for the genetic basis of carbapenemase activity in the other 52 isolates is ongoing.

**Figure 2:**
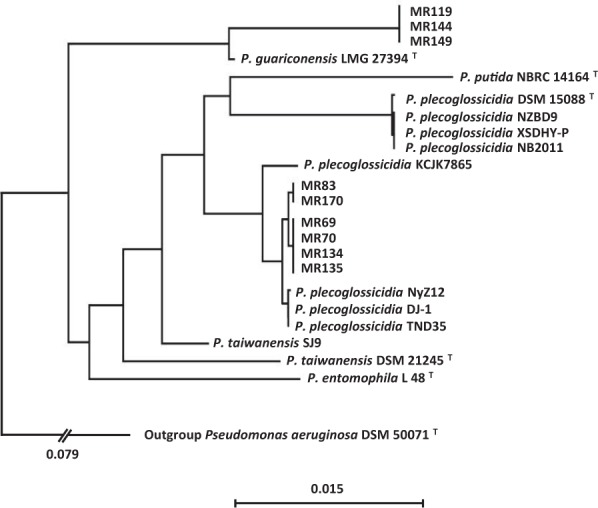
Phylogenetic tree of *bla*_VIM-5_-carrying isolates from Nigerian wetlands and selected members of *Pseudomonas putida* group isolated from fish in Asia (DSM 15088, NZBD9, XDHY-P, NB2011 andDJ-1), soil/sediment in USA (KCJK7865), China (NyZ12) and India (TND35). The tree is based on MLSA of 520 single copy core gene products. Bootstrap support was >90% at all nodes (1000 permutations). Sequence distance is indicated by the horizontal bar.

In order to investigate the genetic background of the nine *bla*_VIM-5_-positive strains, their genomes were sequenced. Genome characteristics as well as isolation dates and origins of the isolates are provided in Supplementary Table S1. The strains were identified via complete 16S rRNA gene sequence comparison and Multi Locus Sequence Analysis (MLSA) of 520 single copy core gene products as *Pseudomonas plecoglossicida* and *Pseudomonas guariconensis* (Fig. 2); members of the *P*. *putida* group^32,33^. Strains identified as *P*. *plecoglossicida* clustered into two groups (MR69, MR70, MR134 and MR135 from AW, and MR83, MR170 from AP). Whole genome-wide SNP counts ranged from 19 to 55 for the most closely related strains, showing that the strains are not identical (Fig. S1).

### *bla*_VIM-5_ was present on 3 different class 1 integrons structures in the isolates

**Figure 3:**
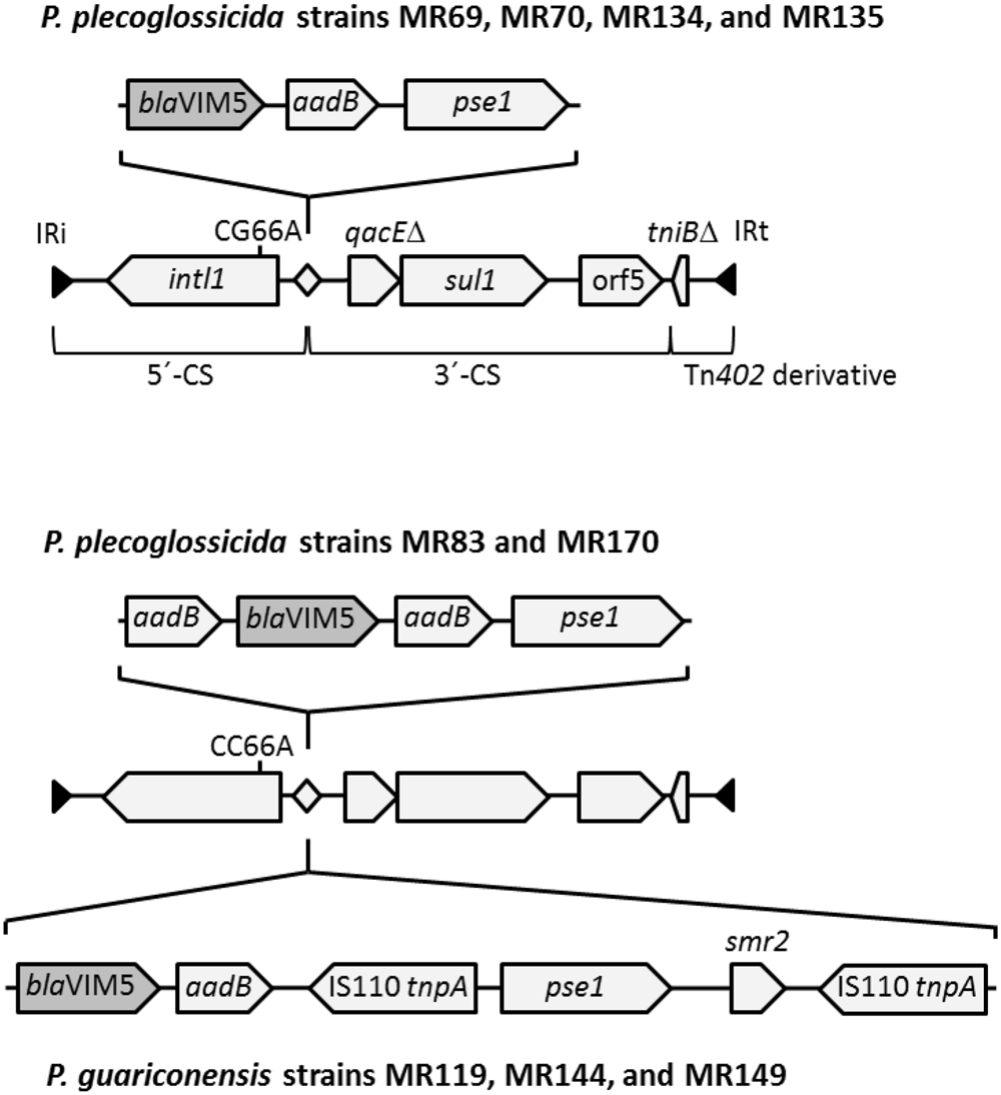
Features of class 1 integrons carrying the carbapenemase gene *bla*_VIM-5_ in members of the *Pseudomonas putida* group isolated from Nigerian wetlands. The range of the canonical 5′-CS, 3′-CS, and defective Tn*402* derivative are indicated by brackets in the depiction for strains MR69, MR70, MR134, and MR135. The integrons were bordered by 25 bp-long canonical inverted repeats IRi and IRt, indicated by filled triangles. Besides one non-synonymous SNP at nt position 66 of *intl1*, the integron backbones were 100% identical. The *attl1* site at which the antibiotic resistance gene cassettes were inserted is indicated with a diamond.

Analysis of the genomes revealed the presence of *bla*_VIM-5_ on Tn402-like class 1 integrons with canonical 5′CS and 3′CS, defective transposition module, and three different gene cassette contents and arrangements (Fig. 3). All the *bla*_VIM-5_-bearing integrons contained the aminoglycoside adenyltransferase gene *aadB* sharing 100% sequence identity with *aadB* from *P*. *aeruginosa* (accession number JF412714)^34^ and the carbenicillin-hydrolysing class A beta-lactamase gene *bla*_PSE-1_ sharing 100% sequence identity with *bla*_PSE-1_ from *P*. *aeruginosa* RIVM-EMC2982 (accession number CP016955). In *P*. *plecoglossicida* strains MR69, MR70, MR134 and MR135 the integron harboured the cassettes *aadB*|*bla*_VIM-5_|*bla*_PSE-1_. In *P*. *plecoglossicida* strains MR83 and MR170 the integron contained a second *aadB* copy in the order *aadB*|*bla*_VIM-5_|*aadB*|*bla*_PSE-1_. In *P*. *guariconensis* strains MR119, MR144, and MR149 the integron carried two identical copies of *tnpA*, encoding for a transposase of the IS116/IS110/IS902 family with 99% sequence identity with transposase of *P*. *aeruginosa* (accession number EF614235)^35^, as well as *smr2*, encoding for a small multidrug resistance efflux protein sharing 75% sequence identity with *smr2* from *P*. *aeruginosa* (accession number KC776907) in the order *bla*_VIM-5_|*aadB*|*tnpA*|*bla*_PSE-1_|*smr2*|*tnpA*. Based on sequence analysis of the integrase genes, the gene cassettes on the integrons were under the control of weak promoters PcW and a second inactive promoter P2. Weak promoters usually have strong integrase excision activity^36^.

In *P*. *plecoglossicida* strains MR69, MR70, MR134 and MR135, the *bla*_VIM-5_-bearing integron was part of 491 kb-large chromosomal contigs. At both integron ends, defined by canonical 25 bp-long inverted repeats IRi and IRt, there were identical copies of an IS6 family transposase IS6100 followed by a second IRt copy. At the 5′-CS side of the integron there was another integron which lacked *bla*_VIM-5_ but was otherwise 100% identical with the *bla*_VIM-5_-bearing one. At the Tn402-derivative side, the IS6-type transposase gene was adjacent to a 2.3 kb-long sequence with 100% nucleotide sequence identity to the adjacent region of class 1 integrons on the large plasmids pBM413 (from *P. aeruginosa* strain PA121617; accession number CP016215) and pSY153-MDR (from *P*. *putida* strain SY153, accession number KY883660). The 4 genes within these 2.3 kb region encode for hypothetical proteins conserved in *Pseudomonas* and some other Gammaproteobacteria. Following those genes was a 477 kb-large region wich had high synteny with a segment in the chromosome of *P*. *putida* KT2440 (accession number AE015451). For the other sequenced strains the genomic locus of the *bla*_VIM-5_ integrons could not be resolved and identical integrons without *bla*_VIM-5_ were not detected. However, none of the contigs appeared to be derived from plasmids. All sequenced strains also carried a Tn402*-*like class 1 integron with *bcr2*, encoding for bicyclomycin resistance protein as sole gene cassette.

### Resistance phenotypes and additional resistance genes identified via WGS

In addition to their various phylogenetic affiliations with the *P*. *putida* group, the reaction of the nine isolates to antibiotics used in determination of minimum inhibitory concentration (MIC) differed (Table 1). All isolates showed resistance to the highest concentration of imipenem (IMP) and sulphamethoxazole (SMX), and the six *P*. *plecoglossicida* isolates were resistant to ciprofloxacin (CIP). The MICs of aztreonam (ATM) for MR70 and MR134 as well as the MICs of ceftazidime (CAZ) for MR83, MR170, MR119 and MR149 were different for each isolate, further confirming that the strains were not identical.

**Table 1:** Antibiotic resistance phenotype and resistance genes profile of the 9 *bla*_VIM-5_-producing isolates from the Nigerian wetlands.

Strain	MIC (mg/L)^a^					Additional Genes Detected by WGS Analysis
	IMP	ATM	CAZ	CIP	SMX	
*Pseudomonas plecoglossicida* MR69	>32	48	>256	>32	>1024	*aph(3*″*)-lb*, *aph(6*″*)-id*, *ampC*, *macAB*, *tetC*, *gyrA* mutation
*P*. *plecoglossicida* MR70	>32	128	>256	>32	>1024	*aph(3*″*)-lb*, *aph(6*″*)-id*, *ampC*, *macAB*, *tetC*, *gyrA* mutation
*P*. *plecoglossicida* MR134	>32	64	>256	>32	>1024	*aph(3*″*)-lb*, *aph(6*″*)-id*, *ampC*, *macAB*, *tetC*, *gyrA* mutation
*P*. *plecoglossicida* MR135	>32	48	128	>32	>1024	*aph(3*″*)-lb*, *aph(6*″*)-id*, *ampC*, *macAB*, *tetC*, *gyrA* mutation
*P*. *plecoglossicida* MR83	>32	16	96	>32	>1024	*aph(3*″*)-lb*, *aph(6*″*)-id*, *ampC*, *macAB*, *tetC*, *gyrA* mutation
*P*. *plecoglossicida* MR170	>32	16	>256	>32	>1024	*aph(3*″*)-lb*, *aph(6*″*)-id*, *ampC*, *macAB*, *tetC*, *gyrA* mutation
*P*. *guariconensis* MR119	>32	24	32	0.25	>1024	*aph(3*″*)-lb*, *aph(6*″*)-id*, *ampC*, *macAB*, *tetB*, *tetC*, *tetG*, *floR*
*P*. *guariconensis* MR144	>32	32	32	0.25	>1024	*aph(3*″*)-lb*, *aph(6*″*)-id*, *ampC*, *macAB*, *tetB*, *tetC*, *tetG*, *floR*
*P*. *guariconensis* MR149	>32	16	>256	0.38	>1024	*aph(3*″*)-lb*, *aph(6*″*)-id*, *ampC*, *macAB*, *tetB*, *tetC*, *tetG*, *floR*

^a^Antimicrobials abbreviations and test concentrations. IMP: Imipenem; ATM: Aztreonam; CAZ: Ceftazidime; CIP: Ciprofloxacin; SMX: Sulfamethoxazole; 0.002–32 mg/L (IMP and CIP), 0.016–256 mg/L (ATM and CAZ), 0.064–1024 mg/L (SMX).

Ten additional antibiotic resistance genes were identified in the genomes of the strains conferring resistance to six classes of antibiotics (Table 1). The *aph(3*′*)-ib*, *aph(6*′*)-Id*, *macAB*, *tetC* and *ampC* were common to all strains while the *tetB*, *tetG*,and *floR* were detected exclusively in *P*. *guariconensis* MR119, MR144 and MR149. A *gyrA* mutation (Thr83Ile) conferring fluoroquinolone resistance was present in all the *P*. *plecoglossicida* strains but not in the *P*. *guariconensis* isolates, consistent with the sensitivity of the latter strains to ciprofloxacin.

## Discussion

This study reports the occurrence and genetic environment of *bla*_VIM-5_ in bacteria isolated from four Nigerian wetlands. Ultimately, this is no suprise given the widespread pollution of aquatic ecosystems in Nigeria. The detection of the *bla*_VIM-5_ gene in closely related members of the *P*. *putida* group suggests an establishment of these species as reservoir of *bla*_VIM_ in the two wetland ecosystems. Members of the *P*. *putida* group are typically found in the environment but have also been reported as important opportunistic pathogens^37^ and reservoir-hosts of metallo-beta-lactamases^38^.

Previously, reports of *bla*_VIM-5_ have only been limited to clinical bacterial isolates from the Asian and African continents being identified in clinical isolates of *P*. *aeruginosa*, *K*. *pneumoniae*, *Serratia marcescens*, *Enterobacter cloacae* and *Proteus mirabilis* from Turkey^27,39–41^, Thailand^27^, Nigeria^27^, India^42^ and China (unpublished, GenBank Accession Number KM589496). This however, is the first report of *bla*_VIM-5_ in environmental bacterial isolates. There is no information available on the gene context of the *bla*_VIM-5_ detected in the Nigerian clinical isolates. However, much like we observed in this study, the *bla*_VIM-5_ in the clinical isolates reported in some of the aforementioned studies^39,41,42^ and the unpublished Chinese isolate are located on class 1 integrons. Interestingly, in all the clinical isolates, the *bla*_VIM-5_ occurred as the first cassette on the integrons, while in six of our environmental isolates (MR69, MR70, MR83, MR134, MR135 and MR170), the gene occurred as the second cassette suggesting different evolutionary pathways for the *bla*_VIM-5_–bearing class 1 integron in the clinical and environmental isolates.

The localisation of *bla*_VIM-5_ within three novel integron structures in two species of *Pseudomonas* recovered from two wetlands points to a high propensity for mobilisation of this gene to other hosts within and outside the wetland ecosystem. This propensity is underscored by the involvement of weak promoters PcW and inactive second promoters P2 in the control of this gene and the associated gene cassettes in all isolates. A previous study has reported an inverse correlation between promoter strength and excision activity^36^. Similar to this observation, the *bla*_VIM-5_ on the class 1 integron of *E*. *cloacae* SMART 28, a clinical isolate from Turkey is also under the control of a weak promoter PcW^41^. Further, the genome sequences of MR69, MR70, MR134 and MR135 revealed the presence of an additional integron which is otherwise 100% identical to the *bla*_VIM-5_–bearing integron except for the absence of *bla*_VIM-5_, suggesting that the acquisition of the *bla*_VIM-5_ cassette was a recent event. It also appears that the capturing of the *bla*_VIM-5_ cassette by the integrons of all sequenced strains occurred via three independent events judging from the localisation of the cassette in three novel integron structures.

Reports of carbapenemase genes in bacteria from environmental sources in Nigeria are not available for a comparative assessment of our results. However, the frequency of carbapenemase genes detection in this study is slightly higher than the prevalence rates of 5.5% and 10.2% reported in two of the hospital-based studies carried out in Nigeria^22,23^. Further, while we detected only *bla*_VIM_, these two Nigerian studies detected other carbapenemase genes in addition to *bla*_VIM_ and the carriage of multiple carbapenemase genes among the investigated CRB. The three independent Nigerian studies are however similar in one respect: the low detection frequency of known carbapenemase genes among the CRB tested. This might be an indication of the presence of novel gene(s) or gene variants in these strains, or the mediation of carbapenem resistance by intrinsic carbapenemase genes^43–45^.

To conclude, we isolated bacteria carrying the MBL gene *bla*_VIM-5_ in members of the *P*. *putida* group in two polluted wetlands in southwestern Nigeria. The link between the study sites and the city’s water supply system heightens the public health risk associated with this observation as this, together with the association of the gene with seemingly active mobile genetic elements, increases the chance of dissemination of the gene to the human population through the water-human route. There is therefore an urgent need for further studies aimed at assessing the actual distribution and persistence of CRB and corresponding carbapenemase genes in the Nigerian aquatic ecosystems.

## Materials and Methods

### Sampling sites and sample collection

Four wetlands in Ibadan and Lagos, located in south-western Nigeria (Fig. 1), were selected as sampling sites. In Ibadan, Awba (AW) wetland (07.4468°N, 03.8763°E) is within a university campus and receives untreated wastewater and sewage from at least five of the university’s hostel facilities, the university’s fish farm and the zoological garden. Apete (AP) wetland (07.4577°N, 03.8828°E) is located behind the campus of a polytechnic and receives domestic wastewater from a network of sources including hostel facilities of the polytechnic and seepages from a solid waste dump site. Water from AW drains into Awba Dam, which serves as the university’s water reservoir. Awba Dam and AP drain into Eleyele Lake, which is the water source for about half of Ibadan’s population of 3.5 million residents. In Lagos, Abule Agege (AA) (06.5145°N, 03.4002°E) is a large expanse of mangrove wetland located within a university campus. The wetland has a direct link to the Lagos Lagoon and is polluted from several non-point sources including untreated sewage and wastewater from a network of hostel facilities of the university and direct discharge of wastewater from freshwater aquaculture. Ogbe Creek (OC) (06.5135°N, 03.3937°E) runs through a mangrove swamp within the campus of the same university as AA. Its primary source of pollution is seepages from a solid waste dump site located upland about 300 meters from the creek. Triplicate sediment samples were collected monthly from October 2014 through January 2015 from the upper 1 cm portion of each wetland. The samples were pooled together and stored at −80 °C until processed for isolation of bacteria.

### Bacteria isolation

CRB were isolated from the sediment samples by selective enrichment on MH agar, EMB agar, and PI agar, all supplemented with 4 mg/L meropenem (Glentham Life Sciences, Corsham, UK) as described^12^, except that saline suspensions (25 g sediment in 225 mL 0.85% saline) of pooled triplicate samples were inoculated into tryptone soy broth (TSB) supplemented with meropenem (4 mg/L) and the enriched TSB cultures (200 µl each) were plated on selective EMB, PI and MH agar plates. Further, all incubations took place at 35 °C. All carbapenem-resistant isolates representing different colony morphotypes based on size, colour, surface texture and colony edge were selected from the plates, subcultured on selective MH plates and stored in 15% (w/v) glycerol at −80 °C.

### Identification of carbapenem-resistant bacteria

Total genomic DNA was extracted (DNeasy blood and Tissue Kit, Qiagen) and used as template in PCR with universal primers 27F^46^ and 1378R^47^ targeting the bacterial 16 S rRNA gene. Amplicons were purified (GeneJet PCR Purification Kit, Thermo Fisher Scientific), sequenced from both ends (Macrogen Europe, Amsterdam) and obtained sequences were used in BLASTn searches (https://blast.ncbi.nlm.nih.gov/Blast) for phylogenetic assignment of the isolates.

### Phenotypic detection of carbapenemase production, and PCR survey of carbapenemase and other beta-lactamase genes

Phenotypic detection of carbapenemase activity was carried out by the Blue-Carba Test^48^. Surveys for carbapenemase genes (*bla*_VIM_, *bla*_KPC/BIC_, *bla*_NDM_, *bla*_IMP_, *bla*_SPM_, *bla*_AIM_, *bla*_DIM_) were carried out via multiplex PCR with primers and conditions previously described^49^ using Kappa 2G Fast Multiplex PCR Mix (KAPA Biosystems, Boston Massachusetts, USA). Multiplex PCR was also used to search for other beta-lactamases (*bla*_TEM_, *bla*_SHV_, *bla*_CTX-M_, *bla*_VEB_, *bla*_GES_, *bla*_PER_, *bla*_OXA-1_, *bla*_OXA-2_, *bla*_OXA-10_, *bla*_OXA-23_, *bla*_OXA-24_, *bla*_OXA-48_, *bla*_OXA-51_, *bla*_OXA-58_, *bla*_CMY-1_, *bla*_CMY-2_, *bla*_ACC_, *bla*_ACT_, *bla*_DHA_ and *bla*_FOX_) with primers and conditions previously described^49,50^. *Pseudomonas aeruginosa* 11 (*bla*_VIM*-*2_), *P*. *aeruginosa* 4 (*bla*_IMP*-*15_), *Klebsiella pneumonia*e ST512 (*bla*_KPC-13_), *K*. *pneumoniae* Mnasey (*bla*_TEM-1_, *bla*_SHV*-*11_, *bla*_CTX-M-15_, *bla*_NDM-1_), *Acinetobacter baumannii* AYE (*bla*_VEB_), and *Providencia rettgeri* H1736 (*bla*_NDM-1_) were used as positive controls. The correct amplification of any detected gene was confirmed with single primer pairs using DreamTaq Green Master mix (Thermo Fisher Scientific) as reported previously^51^. Amplicons were purified and sequenced from both ends with respective primers used in PCR (GATC, Konstanz, Germany).

### MIC determination

The MICs of imipenem (IMP), aztreonam (ATM), ceftazidime (CAZ), ciprofloxacin (CIP) and sulfamethoxazole (SMX) were determined by E-test (Liofilchem Diagnostici, Italy) on MH agar plates according to the manufacturer’s instructions. MH agar plates were inoculated with standardised (0.5 McFarland Standard) saline suspension of each isolate prepared from overnight cultures on MH agar plates. E-Test strips were carefully layered on each inoculated plate and plates were incubated at 35 °C for 18 hrs. Results were interpreted with the CLSI MIC interpretive criteria for *P*. *aeruginosa*^52^.

### WGS and analyses of blaVIM containing isolates

Between 500–1000 ng genomic DNA of each isolate was sheared with a Covaris S220 sonication device (Covaris Inc.; Massachusetts, USA) with the following settings: 55 s, 175 W, 5% Duty factor, 200 cycles of burst, 55.5 μL input volume. Sequencing libraries were prepared by using the NEBNext® Ultra™ DNA Library Prep Kit for Illumina® (New England Biolabs, Frankfurt, Germany) as per the manufacturer’s instructions. The libraries were sequenced with an Illumina® MiSeq machine using v3 chemistry and paired-end approaches with 76 cycles (strains MR69, MR70, MR83, MR119, MR144, MR170) and/or 301 cycles (strains MR69, MR83, MR119, MR134, MR135, MR149) per read. Raw sequences were subjected to adapter clipping and quality trimming using Trimmomatic^53^, and processed reads were assembled with SPAdes v3.6.2^54^. Assembly quality and taxonomic placement of the genome were assessed with CheckM v1.0.4^55^. Prokka v1.11 was used for automated annotations^56^. Antibiotic resistance gene markers were searched for manually and via the Comprehensive Antibiotic Resistance Database (CARD)^57^. In strains MR69, MR70, MR134 and MR135 the entire *bla*_VIM_-carrying integron as well as the linkages with the large chromosomal segment and the adjacent integron without *bla*_VIM_ were Sanger sequenced. In the other strains PCR-mapping and Sanger sequencing were used for scaffolding of the gene cassettes.

Orthologs shared by the different isolates as well as selected references were determined using the bidirectional BLAST+ approach implemented in Proteinortho 5^58^. In order to determine the phylogenetic relationships between the isolates and references via Multi Locus Sequence Analysis (MLSA) with maximum resolution, the single copy core genome of all comparison genomes was determined, excluding all genes with duplicates or missing orthologs in any comparison genome. After alignment with MUSCLE^59^, the gene products were concatenated and un-alignable regions were filtered out using gblocks^60^. The remaining aligned conserved core genome product amino acid residues (187,324 positions) were subjected to phylogenetic clustering using the Neighbor Joining algorithm with 1000 bootstrap permutations. Whole genome SNP analysis was carried out in CLC Genomics Workbench 11.0.1.

## Electronic supplementary material

Supplementary Information

## Data Availability

The 16S rRNA gene sequences of the isolated CRB are available in DDBJ/ENA/GenBank under accession numbers MG674312-MG674376. The WGS projects have been deposited at DDBJ/ENA/GenBank under the accession numbers PJCJ00000000 - PJCR00000000.
