# Supplementary material for: Detection of the carbapenemase gene blaVIM-5 in members of the Pseudomonas putida group isolated from polluted Nigerian wetlands
Source: Sci Rep. 2018 Oct 11;8:15116. doi: 10.1038/s41598-018-33535-3 (PMC6181998; doi:10.1038/s41598-018-33535-3)
Supplement: Supplementary file 1 — Supplementary Information [file 41598_2018_33535_MOESM1_ESM.doc]

**Detection of the carbapenemase gene *bla*_VIM-5_ in members of the *Pseudomonas putida* group isolated from polluted Nigerian wetlands**

Olawale O. Adelowo, John Vollmers, Ines Mäusezahl, Anne-Kristin Kaster and Jochen A. Müller

**Supplementary Table S1**. Genome characteristics, isolation source and dates of 9 members of the *Pseudomonas putida* group from Nigerian wetlands

|  | ***Pseudomonas plecoglossicida*** | | | | | | ***Pseudomonas guariconensis*** | | |
| --- | --- | --- | --- | --- | --- | --- | --- | --- | --- |
|  | **MR69** | **MR70** | **MR134** | **MR135** | **MR83** | **MR170** | **MR119** | **MR144** | **MR149** |
| **Isolation source** | AW | AW | AW | AW | AP | AP | AP | AP | AP |
| **Isolation date** | Oct 2014 | Oct 2014 | Oct 2014 | Oct 2014 | Nov 2014 | Nov 2014 | Oct 2014 | Oct 2014 | Oct 2014 |
| **Total contig count [No]** | 73 | 69 | 86 | 95 | 70 | 81 | 89 | 84 | 88 |
| **Total length [bp]** | 5,882,313 | 5,872,103 | 5,884,059 | 5,878,779 | 5,817,368 | 5,806,378 | 5,616,648 | 5,614,718 | 5,617,362 |
| **Largest contig length [bp]** | 686,561 | 530,793 | 670,695 | 491,336 | 572,982 | 320,627 | 549,660 | 549,615 | 549,660 |
| **N50 [bp]** | 258,357 | 185,194 | 186,762 | 150,274 | 158,785 | 148,407 | 210,549 | 210,526 | 246,969 |
| **L50 [No]** | 7 | 10 | 10 | 12 | 12 | 13 | 9 | 10 | 8 |
| **N80 [bp]** | 97.142 | 94.696 | 90.672 | 67.615 | 85.422 | 77.575 | 117.109 | 114.270 | 119.304 |
| **L80 [No]** | 20 | 24 | 22 | 31 | 27 | 30 | 20 | 22 | 19 |
| **Theoretical coverage [x]** | 90 | 35 | 42 | 24 | 59 | 16 | 120 | 61 | 59 |
| **GC content [%]** | 62.49920345 | 62.50081271 | 62.49705049 | 62.502706 | 62.62053821 | 62.62261533 | 62.22444824 | 62.22438623 | 62.22420342 |
| **Completeness** | 99.83 | 99.83 | 99.83 | 99.83 | 99.87 | 99.82 | 100 | 100 | 100 |
| **Contamination** | 1.51 | 1.51 | 1.51 | 1.51 | 0.67 | 0.67 | 0.62 | 0.62 | 0.62 |
| **Heterogology** | 0 | 0 | 0 | 0 | 0 | 0 | 0 | 0 | 0 |

*Notes*:

AW=Awba wetland in Ibadan, Nigeria

AP =Apete wetland in Ibadan, Nigeria

N50=smallest contig of the size-sorted contigs that make up at least 50% of the respective assembly

L50=number of contigs that make up at least 50% of the respective total assembly length

N80=smallest contig of the size-ordered contigs that make up at least 80% of the respective assembly

L80=number of contigs that make up at least 80% of the respective total assembly length

Completeness=Fraction [%] of expected unique universal marker genes that could be identified

Contamination=Fraction [%] of indentified universal marker genes that occur in multiple copy number (does not necessarily indicate actual contamination)

Heterology=Fraction [%] of putative multicopy marker genes with more than 90% amino acid identity between the respective copies

**MR83**

**MR170**

**MR69**

**MR70**

**MR134**

**MR135**

**0.386**

**0.00001**

**Fig. S1**: SNP trees of *bla*_VIM-5_-carrying isolates affiliated with *Pseudomonas guariconensis* **(Panel A)** and *Pseudomonas plecoglossicidia* **(Panel B)**. The complete genomes of *P. guariconensis* LMG 27394 (Assembly: GCF_900102675.1) and *P. plecoglossicidia* NyZ12 (Li et al. 2015) were used as reference. There were 20 SNP between MR119 and MR149, 39 SNP between MR119 and MR144, 19 SNP between MR83 and MR170, 24 SNP between MR69 and MR70, 22 SNP between MR134 and MR135, and 55 SNP between MR70 and MR134. Bootstrap support was >90% at all nodes (1000 permutations). Sequence distance is indicated by the horizontal bar.

Li, X., Li, C. Z., Mao, L. Q., Yan, D. Z., & Zhou, N. Y. (2015). Complete genome sequence of the cyclohexylamine-degrading *Pseudomonas plecoglossicida* NyZ12. *Journal of biotechnology*, *199*, 29-30.

**MR144**

**MR119**

**MR149**

**A**

**B**
